# May-Thurner Syndrome: A Case Report and Review of the Literature

**DOI:** 10.1155/2013/740182

**Published:** 2013-02-20

**Authors:** Shivani Kalu, Payal Shah, Aparna Natarajan, Nwabundo Nwankwo, Usman Mustafa, Nasir Hussain

**Affiliations:** Department of Internal Medicine, Saint Joseph Hospital, Resurrection Health Care, 2900 North Lake Shore Drive, Chicago, IL 60657, USA

## Abstract

May-Thurner syndrome (MTS) has been recognized as a clinical entity for almost six decades. The true incidence rate of MTS is unknown and perhaps ranges from 22 to 32% according to the autopsy studies in the early twentieth century. However, MTS related deep venous thrombosis (DVT) accounts for only 2%-3% of all lower limb DVTS. In MTS, the left common iliac vein is compressed against the fifth lumbar vertebrae by the right common iliac artery, as it crosses in front of the vein. Chronic pulsation of the artery is thought to cause elastin, collagen deposition, and intimal fibrosis leading to formation of venous spur and venous thrombosis. MTS can present as acute or chronic DVT leading to pulmonary embolism (PE), chronic leg pain, chronic ulcers, or skin pigmentation changes. In this case report we have described an interesting case of a 28-year-old Caucasian female who presented for evaluation of shortness of breath (SOB) associated with cough for one week. SOB was found to be secondary to massive bilateral pulmonary embolism resulting from extensive MTS related DVT of the left lower extremity. Patient underwent pharmacomechanical treatment with local thrombolysis, thrombectomy, and venoplasty along with stent placement that extended to inferior vena caval junction. Subsequently patient was discharged on coumadin. MTS should be considered in differentials when faced with a case of unilateral DVT particularly in younger age group.

## 1. Introduction

Virchow in 1851 observed that iliofemoral deep venous thrombosis (DVT) was five times more likely to occur in the left leg as compared to the right. In 1957, May and Thurner provided an explanation of this phenomenon by discovering an anatomical variation of left common iliac vein. They found that the left common iliac vein had a vascular thickening at the point where it was crossed and compressed against the fifth lumbar vertebrae by overlying right common iliac artery [1]. They called this lesion, “a venous spur” and postulated that a chronic pulsation of the overlying iliac artery is responsible for formation of this spur and that it is the spur, that leads to a venous obstruction. This anatomic variant later became widely recognized as the May-Thurner syndrome (MTS).

In this paper we have described an interesting case of a young female patient who presented with worsening acute shortness of breath due to massive bilateral pulmonary embolism resulting from MTS related extensive DVT of the left leg. 

## 2. Case Presentation 

**Figure 1:**
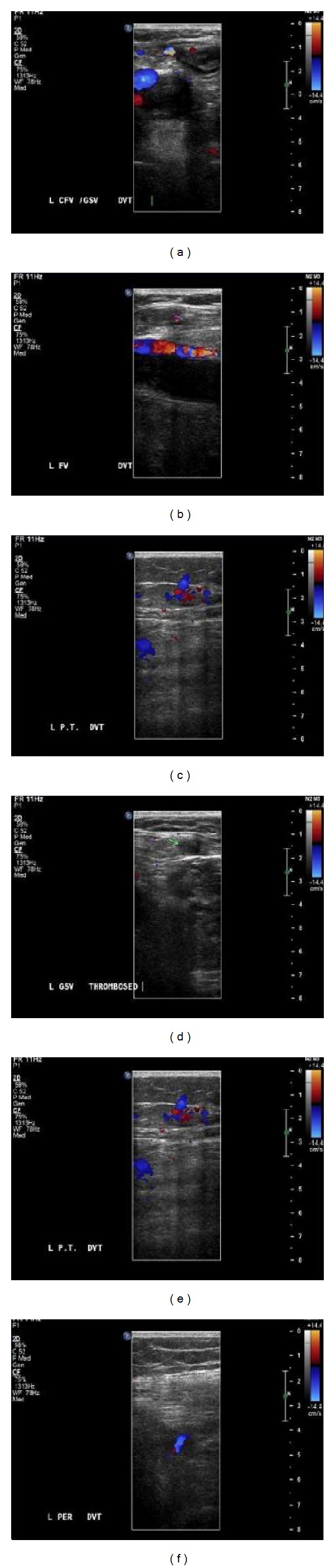
The left common femoral (a); left femoral (b); left popliteal (c); left great saphenous (d); left posterior tibial (e); left peroneal veins (f) demonstrate extensive thrombosis, loss of flow on Doppler. Veins also demonstrate an increase in the size and noncompressibility with ultrasound probe which are consistent with DVT.

**Figure 2:**
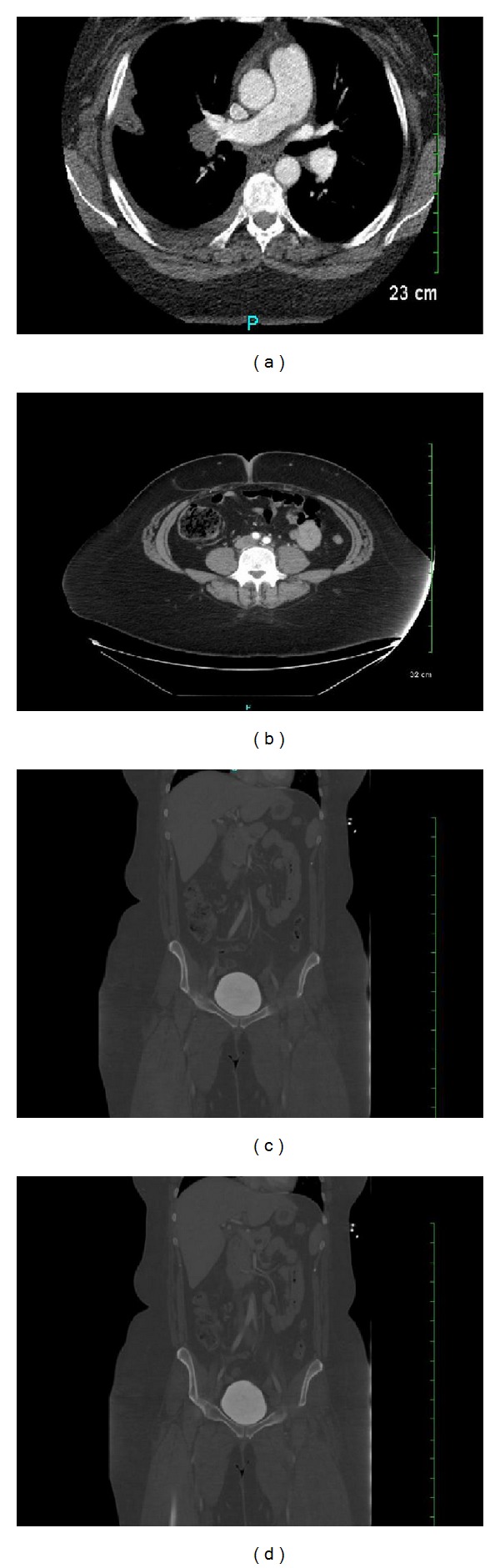
Massive bilateral PE noted in (a), right common iliac artery crossing in front of left common iliac vein noted in (b), (c), and (d), and features consistent with the May-Thurner syndrome.

**Figure 3:**
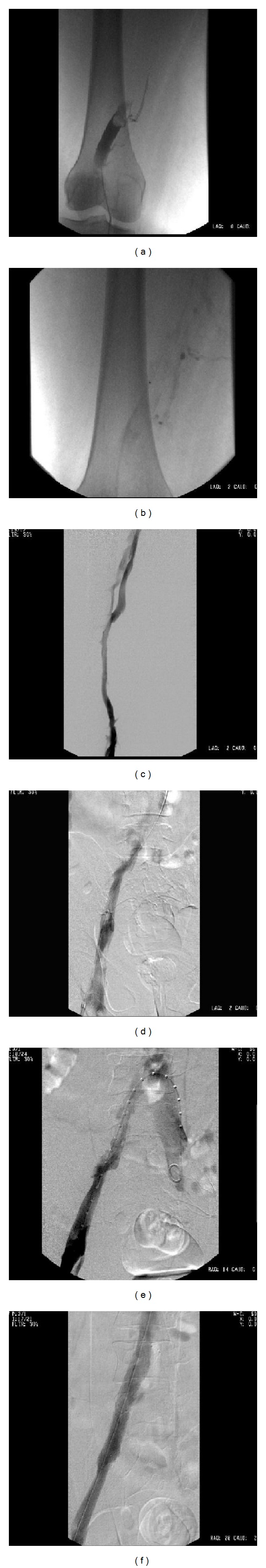
Venography at the day of presentation shows complete thrombotic occlusion of the veins in left leg, dye is not passing beyond popliteal vein ((a) and (b)), (c) demonstrates duplication of the femoral vein. (d), (e), and (f) demonstrate venography at day 3 before and after stent placement, respectively.

A 28-year-old Caucasian female with a past medical history of hypothyroidism presented for evaluation of a new onset shortness of breath associated with cough productive of whitish-colored sputum for one week. Few days prior to the presentation, patient visited another medical facility for same complaints where she was diagnosed with community acquired pneumonia for which antibiotics were given. Symptoms progressively worsened despite of being on antibiotics. On the night of presentation patient woke up suddenly due to acute worsening of breathing difficulty. Patient also noted acute worsening of left leg swelling, and that swelling had become painful. Rest of review of symptoms was significant for history of chest tightness for few hours, leg swelling for last two days, exertional dyspnea and nonspecific myalgia for one week. There was no history of sick contacts, recent travel, trauma, fever, or chills. Patient had never smoked prior to presentation. There was no history of prior clotting problem or clotting problem in the family. At the time of presentation patient was taking levothyroxine and birth control pills. Initial vitals at the time of presentation were: blood pressure of 127/68 mm Hg, pulse 92 beats per minute, temperature 98.4 F (36.7°C), respiratory rate of 19 per minute with an oxygen saturation of 97% on room air. Patient was 5 feet 4 inches tall and weighed 290 pounds. Physical exam revealed young obese female in no acute distress; lungs were clear to auscultation; heart rhythm was regular, no added heart sound, murmur, rub, or gallop; abdomen was soft with positive bowel sounds. Significant physical examination findings were, tender erythematous swelling of left leg from ankle to the thigh; left calf measured 22 inches, whereas right calf measured 20 inches; pulses were palpable in bilateral lower extremities. Considering a very high probability of DVT and pulmonary embolism (PE) patient was given one-time dose of therapeutic subcutaneous enoxaparin. Basic workup including complete blood count, comprehensive metabolic panel, chest X-ray, and coagulation profile (international normalized ratio, prothrombin time, and activated partial thromboplastin time) was within normal limits. EKG and transthoracic echocardiogram were within normal limits and were negative for right ventricular strain pattern. Lower extremity doppler was significant for extensive thrombosis of left common femoral, femoral, great saphenous, posterior tibial, popliteal, and peroneal vein as shown in Figures 1(a)–1(f). CT scan of chest with intravenous contrast revealed massive bilateral PE (Figure 2(a)). CT scan of abdomen and pelvis with intravenous contrast was done to localize extent of the thrombosis and for clinical suspicion of the MTS. CT abdomen supported the diagnosis of MTS as shown in Figures 2(c) and 2(d) and showed extension of the clot all the way through left common iliac vein (Figure 2(b)) into inferior vena cava (IVC). Subsequently the patient underwent interventional radiology (IR) guided venography (Figures 3(a) and 3(b)) and local thrombolysis with the alteplase followed by continuous infusion of alteplase and heparin through catheter laid across femoral and iliac veins with a closer monitoring of CBC and coagulation profile. On a subsequent day patient underwent IR guided venography revealing duplicate femoral veins (Figure 3(c)), pharmacomechanical thrombolysis, and thrombectomy of left iliofemoral veins. Thrombolysis was done with use of alteplase and angiojet device. Clot was removed with suction thrombectomy using 6 French-catheter and with mechanical thrombectomy using 14 mm × 3 cm balloon resulting in a significant decrease in the clot burden. Alteplase infusion and heparin therapy was continued with close monitoring. On the subsequent day patient underwent repeat venography which showed no significant thrombus burden but revealed narrowing of the left common iliac vein (Figures 3(d) and 3(e)) which confirmed the diagnosis of MTS, venoplasty was done, and two stents made of nitinol (nickel-titanium alloy) measuring 14 mm × 8 cm and 14 mm × 3 cm were deployed in iliocaval junction with a good flow through stent into IVC (Figure 3(f)). On the subsequent day alteplase was discontinued, heparin was held for concerns of heparin induced thrombocytopenia which later was found to be negative on serotonin release assay, anticoagulation was continued with fondaparinux, and coumadin was started. Patient was also started on aspirin indefinitely and clopidogrel for at least six weeks. Subsequently patient did well and was discharged home on coumadin and a bridging dose of fondaparinux with instructions to closely followup with primary care physician. Patient was instructed to continue coumadin at least for six months unless she was found to have any thrombophilia, to discontinue birth control pills, and use an alternative form of contraception. Subsequent hypercoagulable workup excluded diagnosis of protein C, protein S, antithrombin III deficiency, factor V Leiden mutation, prothrombin gene mutation, antiphospholipid antibody syndrome, and hyperhomocysteinemia. 

## 3. Discussion

The true incidence rate of MTS is unknown and perhaps ranges from 22 to 32% according to the autopsy studies in the early twentieth century [1, 2]. Despite of high prevalence of MTS the clinical prevalence of MTS related DVT accounts for only 2%-3% of all lower extremity DVTs [3] and is usually seen in females between the age group of 20–40 years. The trauma from chronic arterial pulsation of the left common iliac artery is believed to cause deposition of elastin and collagen [4] in left common iliac vein resulting in a spur formation. Furthermore continued trauma leads to local extensive intimal proliferation resulting in venous thrombosis and impaired venous return [5]. Many other variants for MTS [6–9] have been described. 

Thorough history, physical examination and diagnostic workup including thrombophilia workup should always be performed to identify the risk factors for DVT when faced with a diagnosis of DVT specifically in younger patients. Patients with known diagnosis of MTS should always have evaluation for thrombophilia, as Kolbel et al. [10] found that 67% of patients with chronic iliac vein occlusion or MTS have some form of thrombophilia. Our patient had classic form of MTS and had no evidence of thrombophilia. 

Uhl et al. [11] demonstrated that truncal venous malformation of the femoral vein may be present in about 12% of the general population. Femoral vein duplication is a common truncal venous malformation; in setting of duplicated femoral vein it is not uncommon to find one limb of femoral vein with the clot, whereas the other limb is entirely patent. Clinically if duplication of the vein is not suspected, diagnosis of the DVT may be missed which will lead to catastrophic complication. If the duplication of femoral vein is found on one side then contralateral side will most likely also have the duplication [11]. Our patient had unilateral left sided duplicated femoral veins but the presentation was classic for DVT which allowed us not to miss the diagnosis. To the best of our knowledge this is the first case reporting coexistence of MTS with duplication of femoral veins in a patient.

Patients with MTS usually present with acute or chronic unilateral left lower extremity swelling and pain. Some patients may present with skin pigmentation changes, chronic leg pain, recurrent skin ulcers [5], or varicose veins. Few cases of iliac vein rupture due to MTS have also been reported [12]. MTS related acute or chronic DVT leading to PE may be presenting symptom of MTS as in our patient. 

CT venography, MR venography, intravenous ultrasound, or conventional venography can be used to confirm diagnosis of MTS in the suspected cases. With simple doppler ultrasound it is very difficult to find pathology in the iliac vessels though there have been few case reports where diagnosis of the MTS was suspected on doppler examination of the iliofemoral vessels [4]. 

MTS is treated only when it is symptomatic. Previously open surgical procedures were done for repair of MTS but with advancement of technology less invasive endovascular repair has gained popularity. The mainstay of treatment is removal of the clot with pharmacomechanical thrombolysis and mechanical thrombectomy to prevent postthrombotic syndrome [13] and to repair the anatomical defect with the use of stents and balloon venoplasty. Following placement of stents patients is treated with anticoagulants for at least six months to prevent in-stent restenosis. Some authors suggest that MTS related DVT patients with a high clot burden should also undergo IVC filter placement [14]. Kwak et al. showed that MTS related DVT patients who had metallic stent placed following thrombectomy had a primary and secondary patency rate of 95% and 100% at a 2-year followup [15]. 

## 4. Conclusion

It is important to consider MTS in differential diagnosis when presented with a case of unilateral DVT especially in a younger age population. If diagnosis is missed, the recurrence of thrombosis, PE, and postthrombotic syndrome will lead to significant morbidity and mortality. The key to successful treatment in MTS related DVT is to fix the anatomical lesion along with removal of the clot and use of anticoagulation. 

Possibility of duplicated femoral vein should always be sought as one may easily miss the clot in the second limb of femoral vein which may lead to catastrophic consequences.
